# The pivotal role of social identification in explaining authentic leadership’s effect on team organizational citizenship behavior

**DOI:** 10.1371/journal.pone.0347874

**Published:** 2026-06-02

**Authors:** Brendan Boyle, Jun Gu, Candy Ying Lu, Zhiming Cheng, Miles Yang, Rebecca J. Mitchell

**Affiliations:** 1 Newcastle Business School, University of Newcastle, Newcastle, Australia; 2 Health at Work Research Centre, Macquarie Business School, Macquarie University, Sydney, Australia; Xi’an Jiaotong-Liverpool University, CHINA

## Abstract

**Purpose:**

The potential for authentic leadership to influence team organizational citizenship behavior (OCB) brings questions regarding the mechanisms underpinning such an effect into focus. Drawing on the social identity approach, we propose and investigate a moderated mediation model that authentic leadership increases team members’ OCB through increased team identification and that this mediated path is contingent on members’ professional identification.

**Methodology:**

We tested our model first using a causality chain design (two recall experiments with 99 and 151 working professionals respectively) and then a multi-source field survey of 343 leaders and team members from 60 healthcare teams.

**Findings:**

We find support for our moderated mediation model confirming that only in multidisciplinary professional teams characterized by weak professional identification among members does authentic leadership enhance member OCB through team identification. These findings allow us to explain ambiguities in the impact of authentic leadership in multidisciplinary professional teams, particularly in critical contexts such as healthcare.

**Originality/Novelty:**

We integrate social identity theory with learning from the sociology of the professions to address important gaps in our knowledge of how professional identification influences intra-team extra-role dynamics across professional boundaries. Our investigation of the mechanisms through which authentic leadership influences OCB represents a particularly novel exploration, as we argue that professional identification can *differentiate* between positive and negative effects of authentic leadership via team identification. The paper’s novelty lies in explicating how professional identification conditions the indirect pathway between authentic leadership and OCB.

## Introduction

Organizational citizenship behavior, defined as discretionary employee behavior that that aims to promote organizational goal achievement [1], has long been argued to enhance organizational dynamics and performance [2]. Indeed, recent research confirms that citizenship behavior enhances performance and effectiveness, both in organizations [3,4] and in teams, the focus of our study [5]. This explains increasing research efforts to understand what predicts citizenship behaviors and the mechanisms explaining these effects [6–8]. One promising area of focus in this endeavor is leadership, with recent efforts that confirm the potential impact of leadership on citizenship behavior [9].

At the same time, the impact and prevalence of unscrupulous leadership has precipitated an upsurge of interest in frameworks of leadership that focus on authenticity, self-regulation and self-awareness [10–12]. Authentic leadership theory has been argued, and evidenced, to promote prosocial behavior and lessen nefarious activity among followers [13–15]. There is also empirical support for the impact of authentic leadership at work, much of which has focused on employee outcomes including motivation, job satisfaction, wellbeing and performance [16,17]. However, though a relationship between authentic leadership and follower citizenship behavior has long been argued, questions have been raised regarding the mechanisms underpinning this effect, particularly at the collective or team level [11]. In addition, ambiguous results continue to emerge (for example, [16,18]), substantiating the current gap in our understanding of authentic leadership’s impact on citizenship behavior which appears neither simple not straightforward. Clarifying the mechanisms of these effects is important given the centrality of both authentic leadership and citizenship behavior in organization and individual wellbeing and performance [2,19,20].

One potentially fruitful avenue in the quest to comprehend why and when authentic leadership influences citizenship lies in social identity theory [21,22]. Theorizing on authentic leadership has argued that such leaders have followers who increasingly identify with their work collective [23,24]. However, while social identification has been demonstrated to significantly influence team dynamics [25], we do not yet understand the explanatory role of social identification in authentic leadership’s influence or, more specifically, its explanatory role in the relationship between authentic leadership and citizenship behavior. We seek to address this unanswered question as, particularly in multidisciplinary teams, our current lack of knowledge presents a substantial challenge, as social identification processes related to both the team and occupation may interact to impact dynamics and performance [22,26,27]. The primacy of identification in understanding team dynamics is echoed in writing on the sociology of the professions, which also highlights the importance of considering both team and professional identification on team dynamics [28,29]. Yet, our understanding of the potential interactive impact of such identification, particularly with respect to citizenship behavior, remains opaque. We thus develop and investigate a mediated moderation model of authentic leadership in multidisciplinary teams, where members’ team identification mediates the relationship between authentic leadership on members’ OCB, contingent on members’ professional identification.

In undertaking this research, we make several important contributions. First, our research contributes to a new understanding of how and when team citizenship behavior emerges. This investigation of the mechanisms through which authentic leadership influences citizenship represents a particularly novel contribution, as we argue that professional identification can *differentiate* between positive and negative effects of authentic leadership via team identification. This investigation also attempts to account for remaining ambiguities in the impact of authentic leadership in multidisciplinary teams, particularly in professional organizations such as healthcare (for example, [30,31]). Further, by focusing on multidisciplinary professional teams we integrate social identity theory with learning from the sociology of the professions to address important gaps in our knowledge of how professional identification influences intra-team citizenship dynamics across professional boundaries.

## Literature review

### Authentic leadership and team identification

Although there have been a wide range of conceptualizations of authentic leadership [32], much recent research and operationalization rests on four primary components [33]: self-awareness, balanced processing, relational transparency and internalized moral perspective [32,34]. Thus, authentic leaders are those who make efforts to develop insight into their traits, behaviors and feelings, to consider information in a balanced (unbiased) manner, to engage followers in an open and transparent way and to reflect on their own values in leadership [35].

Through these behaviors, authentic leadership has been posited to influence the collective social identification of those they lead [36], including team identity an important facet of social identity. The social identity approach, encompassing social identity theory [37] and self-categorization theory [38], provides the conceptual underpinning for this relationship. The social identity approach suggests that team members tend to define themselves and others in terms of their membership of discrete social groups, mainly to reduce uncertainty or enhance oneself [39]. We follow this key theoretical tenet to develop our arguments linking authentic leadership to team identification. First, because team members are motivated to enhance their self-concept and self-esteem, they are more likely to identify with the team if its attributes, values and practices are distinctive and appealing [39,40]. Authentic leaders act in a manner that is consistent with their value and ideals, and also tend to uphold the vision of the organization in which they lead [17,41] as well as their followers [42]. As such, an authentic team leader stands for their own ideals as well as those of the team and broader organization [17]. In this way, authentic leaders provide clarity regarding the team’s place in the organization and the contribution of the team’s work, which has been linked to team identification [39,43,44]. Further, the self-concept-based theory of leadership [45] suggests that authentic leaders may increase the extent to which team membership is perceived as self-enhancing by members. Showing respect for members and commitment to the team’s work conveys that their leader sees the team and its goals as valuable [42], which has also been linked to members’ identification with the team [46].

Recent exploration of leadership factors that promote team identification provides further evidence that authentic leader behaviors are likely to promote such identification [46]. In their exploration of identification-enhancing behaviors, Huettermann et al. [46] found that team identification emerged when leaders provided guidance, encouraged involvement, engaged in role modeling, and actively managed teamwork. Authentic leaders foster member involvement in open and transparent discussion and evaluation of information [47] increases considerate voice and reduces misunderstanding and associated conflict between members [48], and may also contribute to greater clarity of the group prototypical characteristics [49].

Authentic leaders also provide guidance through balanced processing by explicitly weighing up different options and evaluating these in the context of their own moral perspective. Such moral leadership supports team member understanding and appreciation of the significance and purpose of the teams work as well as strengthening team identity [50,51]. By enhancing member perception of the meaning and value of the teams tasks, authentic leaders are able to activate team identification, as members, intrinsically motivated towards a positive self-concept [52], may increasingly value team membership as a source of self-esteem [53].

Finally, authentic leadership is characterized by role modeling [54] with evidence that through role-modelling their personal convictions, authentic leaders build collective commitment to shared team values of transparency, authenticity and integrity [33,55]. Leading by example also contributes to the development of team identification by heightening feelings of belonging and attachment [46]. In conclusion, we predict that authentic leadership with increase team identification as postulated in the following hypothesis:

*Hypothesis 1: Authentic leadership will be positively related to team identification.*

### Team identification and OCB

Research into the impact of a common team identity in a work team has consistently linked it with a reduction in members perception of division between individuals from different social groups and backgrounds [56]. Team identity therefore extends attributions of ‘ingroup’ characteristics, such as honesty, cooperativeness and similarity, to all members of the team. In addition, team members tend to view themselves and each other as personifying the core characteristics of the team. This perceived commonality leads to an increase in the members’ acknowledgement and acceptance of the value and importance of others’ contribution to the team’s work [43,57].

Team members are therefore motivated to offer support and information to other members as their individual identity is closely linked to the team [58,59]. This perception of oneness with the team is reflected in a perception by members that the goals of the team are their own [60] and has previously been linked to helping behavior [61,62]. A shared sense of team identity has been shown to promote members’ tendency to engage in extra-role behaviors that are supportive of the team and its members [27,63], and which enhances the team’s welfare and joint goals [62,64].

*Hypothesis 2: Team identification will be positively related to OCB.*

### Professional identification as a moderator

Despite the argued effect of team identification in promoting collaboration across social categories, there is substantial evidence that team identification cannot completely subsume previous ingroup/outgroup distinctions [65]. This is particularly likely in multidisciplinary professional teams due to the powerful influence of professions and professional identities (e.g., the salience of profession in healthcare settings, [66]). Professional identification reflects perceptions of the self as a member of a defined profession, distinct from, and compared to, other professions [38]. Encompassing a domain of expertise, professional identification also incorporates professionally-based expectations, mindsets and values [67]. As professional identification strengthens, members are more intensely attached to their profession, and the professionally-mandated positions and perspectives are more strongly advocated against other professions’ preferences [43,67].

Team members with strong professional identification are driven more strongly by their professions priorities and preferences, while members with weak professional identification are less motivated to depend on their professions’ preferences [68–70]. In teams comprised of members from the same or similar professional backgrounds, greater professional identification can lead to greater efforts to achieve profession-related priorities in the context of the team’s work [71,26,28]. This is supported by a growing stream of research [28,71,72], and complemented by consistent findings that professional identification interacts with team-level identification to determine team dynamics and performance (for example, [28,29,73,74]).

However, in multidisciplinary professional teams where members come from different professional backgrounds, greater professional identification (i.e., when members are strongly identified with their respective professions) might interfere with team dynamics, and potentially attenuate the connection between team identification and OCB. The impact of team identification in prompting behaviors that support others’ effortsin pursuit of the team’s goal is lessened, and may even be negated, when members are equally driven to pursue their profession’s distinct priorities. Indeed, the strength of team identification has been argued to motivate those with strong professional identification to reassert their distinctiveness and safeguard differentiating aspects of social identity [75,76]. In this situation, strong team identification may engender perceived threat to the distinctive priorities of different member professions [76–78]. Such identity threat has, indeed, been linked to decreased citizenship behaviors [79].

Thus, in multidisciplinary professional teams comprised of members who identify strongly with their respective professions, the perception that team priorities might overshadow those of their profession may prompt members to take actions that undermine the team’s work. Conversely, in multidisciplinary professional teams characterized by comparatively weak professional identities where team members are less driven to pursue the distinct priorities of their profession, team identification is likely to be a more influential determinant of member behavior such as citizenship. This leads to the following hypothesis:

*Hypothesis 3: In multidisciplinary professional teams, professional identification will moderate the relationship between team identification and citizenship behavior, such that the positive relationship between team identification and organizational citizenship behavior is more likely to occur in teams characterized by low professional identification.*

### A moderated mediation model

We have argued that authentic leadership increases team identification. The internalized moral perspective of authentic leaders informs their efforts to ensure balanced processing of information to provide clear guidance and to engage in role modeling of shared team goals and values. In addition, the relational transparency of authentic leaders encourages member involvement, leading to our first hypothesis. We further argued that team identification increases OCB as perception of oneness with the team, and the ingroup status of other team members, motivates efforts to offer support and assistance towards the achievement of the team’s goal. Furthermore, the path between team identity and citizenship is dependent on professional identity: when teams are characterized by strong professional identity, the drive towards supporting other members towards the shared team goal will be diminished, and potentially negated, as member profession more strongly motivates behavior. Conversely, team identification will be more influential when teams are characterized by weak professional identity.

**Fig 1:**
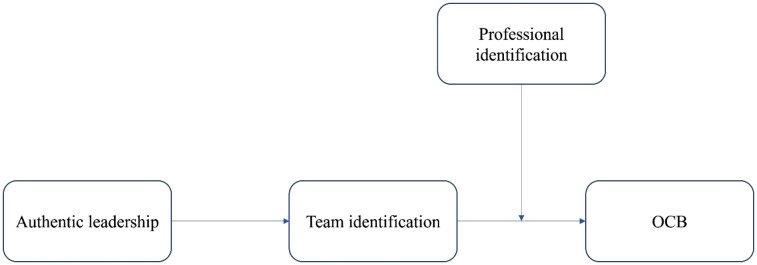
A moderated mediation model of authentic leadership and OCB in multidisciplinary professional teams.

Together, these arguments suggest a moderated mediation model in which team identity mediates the path between authentic leadership and citizenship behavior, contingent on professional identity which leads to our final hypothesis and is depicted in Fig 1.

*Hypothesis 4: Professional identification will moderate the positive mediated relationship between authentic leadership and OCB via team identification, such that the relationship is more likely to occur in teams characterized by low professional identification.*

## Materials and methods

### Research design

The authorship team’s research interest in healthcare teams and professional collaboration was directed to improving understanding and leadership practice in interprofessional healthcare teams. A multi-study design was used to investigate our hypotheses. We used three studies, two experimental recall studies and one field-based survey study, and this use of multiple studies provides confidence in the causal relationships proposed in our theoretical model and the robustness and generalizability of our results beyond the study samples [80]. We first aimed to establish the causal relationships in our model using a causal chain design [81], in which we manipulated the independent variable and the mediating variable in separate experiments (e.g., [82,83]. Specifically, we manipulated authentic leadership to test its effect on team identification in a recall experiment (Study 1) and manipulated team identification to test its interactive effect with professional identification on OCB in a second recall experiment (Study 2). Together these two separate experiments provide stronger causal evidence for our theoretical model than a traditional experimental design where only authentic leadership is manipulated and all other variables are self-reported by participants [81]. Finally, we consolidated the causal chain design with a multi-source field survey of multidisciplinary professional healthcare teams in the US to comprehensively test the moderated mediation model. The questionnaire and methodology for this study was approved by the Human Research Ethics committee of the University of Newcastle (Ethics approval number: H-2013–0346). Data was collected for the two separate studies with the initial study data collected from 21/01/2014 to 24/012015 and the subsequent study data collected from 24/01/2022 to 25/01/2023. Data is available on request in alignment with ethical approval. All participants gave informed implied consent as set out in the participant information statement they were provided with under the ethics approval process. This information statement also conveyed information regarding confidentiality and the anonymity and management of data.

### Study 1: Authentic leadership and team identification

The purpose of Study 1 was to directly test the causal relationship between authentic leadership and employee team identification using a randomized recall experiment. Working professionals from the US were recruited to complete the recall experiment via CloudResearch, a leading participant-sourcing platform formerly known as Turk Prime that helps researchers collect high quality data from Amazon’s Mechanical Turk [84].

#### Sample and procedure.

A total number of 99 working professionals (*M*_age_ = 41.66 years, *SD* = 12.20, 44% female) participated via CloudResearch. This study combined random experimental assignment with the critical incident technique [85], which requires participants to recall real existing events or experiences at work and describe them in detail. To ensure the participants have had previous experience working in multidisciplinary professional teams, we first asked participants to confirm that they have a profession and then asked them to confirm that they have worked in multidisciplinary professional teams led by team leaders. Specifically, we first provided participants with a definition of a profession (“a group of individuals with certain abilities, knowledge, experiences, and values who perform a role in society” and 10 examples (e.g., accountants, dentists, police, pharmacists, etc.), and asked participants to report whether or not they have a profession. The participants who reported not having a profession were disqualified to participate in the study. We then asked participants to report whether they have worked in multidisciplinary professional teams (where members come from two or more different professions) led by team leaders, and those who reported not having such experience were also disqualified. A total number of 114 participants responded to our initial invitation, however 15 of them reported not having a profession or not having any previous experience with multidisciplinary professional teams and were consequently excluded from participating in the recall experiment. The final sample of 99 professionals come from a diversity of industries, such as retail, manufacturing, finance, transportation, government, education, etc.

The participants were randomly assigned to recall and describe either an authentic leader or a non-authentic leader that they had recently worked for as part of a multidisciplinary professional team. They then reported the extent to which they identified with the team led by the leader and the demographics.

**Authentic leadership manipulation:** The participants in the authentic leadership condition were asked to recall a multidisciplinary professional team that they recently worked in, where the team leader demonstrated all the authentic leadership behaviors identified in the 13-item authentic leadership scale developed by [86]. For example, the leader “understands his/her strengths and weaknesses”, “asks for ideas that challenge his/her core beliefs”, and “uses his/her core beliefs to make decisions”. In contrast, the participants in the control condition were asked to recall a team where the leader did not demonstrate authentic leadership. For example, the leader “doesn’t understand his/her strengths and weaknesses”, “doesn’t like ideas that challenge his/her core beliefs”, and “rarely uses his/her core beliefs to make decisions”. All participants successfully recalled a team leader that fits the descriptions in accordance with their experimental conditions (*M*_leader age_ = 42.24 years, *SD* = 10.49, 37.4% female leaders, *M*_team size_ = 11.53 members, *SD* = 13.75, *M*_participant team tenure_ = 17.79 months, *SD* = 24.43).

**Team identification:** On a 7-point Likert scale (1 = *strongly disagree*, 7 = *strongly agree*), participants reported the team identification they experienced toward the team that they just recalled using four items from Mitchell et al. [28], for example, “I felt attached to the team” (*α* = .98).

### Study 2: Team identification, professional identification, and OCB

Consistent with Study 1, we recruited working professionals from the US via CloudResearch to complete a recall experiment to test whether team identification positively influences OCB and whether this effect is stronger among employees with weaker professional identification.

#### Sample and procedure.

A total number of 151 working professionals (*M*_age_ = 37.25, *SD* = 9.89, 41.1% female) participated via CloudResearch. Consistent with Study 1, we combined random experimental assignment with the critical incident technique and required that each participant a) should belong to a profession and b) should have previous experience working in multidisciplinary professional teams led by team leaders. Out of the 182 participants who responded to the initial invitation, 31 were disqualified because they don’t have a profession or because they did not have any experience with multidisciplinary professional teams.

The participants were randomly assigned to recall a multidisciplinary professional team that they recently worked in and that they experienced either strong or weak identification with. They then reported the extent to which they engaged in OCB when working in the team. Finally, the participants reported professional identification, the control variables, and demographics.

**Team identification manipulation:** We developed a team identification manipulation based on Mitchell et al. [28]’s four-item team identification scale. Specifically, the participants in the high team identification condition were asked to recall a multidisciplinary professional team in which they have worked in, where they felt attached to the team, felt a sense of belonging to the team, identified strongly with the team, and saw themselves as part of the team. In contrast, the participants in the low team identification condition were asked to recall a team where they didn’t feel attached to the team, felt no sense of belonging to the team, didn’t identify strongly with the team, and didn’t see themselves as part of the team. All participants successfully recalled a team that fits the descriptions in accordance with their experimental conditions (*M*_team size_ = 12.83 members, *SD* = 13.27, *M*_participant team tenure_ = 12.04 months, *SD* = 22.76, *M*_number of female members_ = 5.46, *SD* = 6.31).

**OCB:** On a 7-point Likert scale (1 = *not at all*, 7 = *a great deal*), the participants reported the extent to which they engaged in OCB when working in the team they just recalled using five items from the Interpersonal Organizational Citizenship Behavior (OCB-I) scale [87–90]. The items (e.g., “going beyond expectations to contribute to the team’s work”) showed high reliability (*α* = .92).

**Professional identification:** On a 7-point Likert scale (1 = *strongly disagree*, 7 = *strongly agree*), the participants responded to 5 items from [28]; e.g., “I feel strong ties with my professional group”; *α* = .91).

**Control variables:** We controlled for participant age and gender in the analyses. Removing these control variables doesn’t change our results. To rule out alternative explanations and to demonstrate that the effect of professional identification goes above and beyond other professional related variables, we also measured and controlled for professional differentiation (i.e., the extent to which participants differentiate themselves from individuals from other professions) and professional salience (i.e., the extent to which profession influences how participants perceive and interact with others). Specifically, on a 7-point Likert scale (1 = strongly disagree, 7 = strongly agree), the participants reported professional differentiation using 4 items (e.g., “Professionals from other professions are NOT part of my ‘in-group’”; *α* = .82) and reported professional salience using 3 items adapted from (2015; e.g., “In a multi-professional team, I often think of team members in terms of their professions”; α = .92)

### Study 3

#### Sample and procedure.

Multidisciplinary professional teams comprised of healthcare workers formed our sample population and teams enrolled in a database hosted by a research services organization formed our sampling frame. We distributed surveys to 210 teams and received responses from 64 teams, representing a 30% response rate. Two independent surveys were distributed to teams: A leader survey was sent to team leaders to collect data on OCB and a member survey was sent to the team members to collect data on our predictor variables. By collecting data from multiple sources we aimed to considerably reduce the risk of methodological issues such as common method bias [91]. In addition, in the member survey, we took additional measures to minimize the risk of common method bias. We varied our anchor labels to disrupt undesirable response patterns and explained to participants that each item in the survey was important and needed to be read and answered carefully [92].

We received an average of five responses per team and used Dawson’s selection rate to assess the extent to which incomplete team responses were sufficient to predict true team scores. The formula for Dawson’s selection rate is *([N – n]/Nn)* with *n* being the number of responses per group and *N* reflecting group size [93]. Dawson’s criteria of.32 was used to assess response rates as this is correlated with true scores at.95 or higher [93]. We removed four teams that did not meet this cutoff, leaving us with 343 participants in 60 teams for analysis. These teams were involved in a range of activities. Almost one-third were established to address complicated patient care problems with the remaining teams tasked with resolving complex clinical and service challenges. A small proportion of teams were involved in the provision of education and training, policy development and research. Consistent with the focus of this paper, all teams were multidisciplinary professional in composition and members included dentists, dieticians, occupational therapists, physiotherapists, pharmacists, medical doctors, registered nurses, psychologists and radiographers. Similar to national proportions, nurses comprised 49% of our sample and 54.5% of US healthcare professionals, however 5% of our participants and 15% of the US healthcare workforce are medical doctors [94]. Teams included seven members on average and had been together for approximately three years and were still working together at the time of data collection. The median age of participants was 40 years, which is similar to the average for US health service employees [95].

**Measures:** We employed intraclass correlations, ICC(1) and ICC(2) and inter-rater reliability, median r_wg_, to justify aggregation [96]. All items were measured on 7-point scales (1 = *strongly disagree*, 7 = *strongly agree*).

*Authentic Leadership* was measured using the Authentic Leadership Inventory [97]. This 14-item scale measures balanced processing, relational transparency, internalized moral perspective and self-awareness and thus fits well with our conceptualization. Example items include “our leader asks for ideas that challenge his/her core beliefs” and “our leader shows that he/she understands his/her strengths and weaknesses” (*α* = .96). The ICC(1) was.19, *F* ratio 2.09, *p* = .000, su*p*porting our claim that team membership accounted for a sizeable part of response variance [98]. The ICC(2) was.52 and the median r_wg_ for authentic leadership was.91, also supporting aggregation to team-level of analysis.

*Team Identification:* Participants completed four items [28]. For example, participants reported the extent to which they “identify strongly with the team” and “see themselves as part of the team” (*α* = .93). Support for aggregation was found as the ICC(1) for this measure was.17, F ratio 1.98, p = .000. The ICC(2) of.50 and median r_wg_ of.86 further supported aggregation.

*Professional Identification:* Same as Study 2. Professional identification is typically conceptualized as an individual construct, however it has also been conceived and operationalized at a team level (for example, [28]), similar to individual characteristics such as personality (for example, [99]). When group-level constructs are measured as individual team member attributes, the resulting data requires aggregation [100]. However, in that case of professional identification, we could not aggregate based on a direct consensus model because we expected some variation between different members and different professions [101]. However, our conceptualization of professional identification was clearly group-level and therefore, Following prior research, we operationalized professional identification with reference to the team’s task [28,99] and determined the method of aggregation using Steiner’s [102]  three categories: disjunctive, conjunctive and additive tasks. In a disjunctive task, the team’s best member determines the team’s performance level, while in a conjunctive task, the team’s weakest member determines output. Typical of multidisciplinary healthcare teams [103] all of our teams were involved in work best categorized as additive, as success necessitated the input of all members with the team’s output being reflective of shared contribution to the team’s task. In alignment with previous research [28], our conceptualization of team-level professional identification reflects an aggregate of the strength of member professional identification. For example, if most members report strong identification with their profession, the team’s score will be high. Though we expected some variation in professional identification within teams, we assessed the extent to which aggregation was justified. The ICC(1) for professional identification was.17, *F* ratio 1.98, *p* = .000. The ICC(2) of.49 and median r_wg_ of.87 also *p*rovided support for aggregation. The Cronbach alpha for professional identity was.91.

*OCB*: We slightly adapted the OCB items used in Study 2 by changing the target of each item to team level. Team leaders rated, for example, the extent to which each team member helped new members get oriented to the team’s task, took on more work to help the team achieve its goals, and went beyond expectations to contribute to the teams’ work (*α* = .96).

*Control Variable:* Professional diversity was included as a control variable following findings that that professional composition may increase innovation [104]. Blau’s [105] index of heterogeneity was used to operationalize diversity: (1-ΣPi^2^), where Pi is the proportion of professionals in *i*th category. Blau’s [105] index is used widely to operationalize job-related and professional diversity [104,106].

## Results

### Results of Study 1

#### Manipulation check.

On a 7-point Likert scale (1 = *strongly disagree*, 7 = *strongly agree*), the participants rated the team leader that they have recalled using 6 items (“authentic”, “transparent”, “self-aware”, “consistent”, “fact-driven” and “openminded”; *α* = .97) developed based on the key authentic leadership dimensions [86]. The participants in the authentic leadership condition reported the leader to have demonstrated authentic leadership to a greater extent (*M* = 5.99, *SD* = .82) compared to those in the control condition (*M* = 2.37, *SD* = 1.45), *t*(97) = 14.95, *p* < .001, supporting the effectiveness of the manipulation.

Descriptive statistics and correlations are presented in Table 1. Because no multi-indicator latent constructs were estimated, CFI/RMSEA are not applicable for this study.

**Table 1:** Descriptive statistics and correlations among key variables in Study 1.

Variable	*M*	*SD*	2	3	4	5	6
1. Authentic leadership	.46	.50	.55^**^	−.08	.03	.04	−.06
2. Team identification	4.53	1.98		−.02	−.04	.04	−.02
3. Leader age	42.24	10.49			−.01	.52^**^	.10
4. Leader gender	.37	.49				.08	.65^**^
5. Participant age	41.66	12.20					.21^*^
6. Participant gender	.44	.50					

Note: ^*^ p < .05, ^**^ p < .01, ^***^ p < .001; authentic leadership (0 = low, 1 = high); gender (0 = male, 1 = female).

#### Team identification.

To test the effect of authentic leadership, we conducted an ANOVA of team identification with authentic leadership as a fixed factor predictor and leader age, leader gender, employee age, and employee gender as covariates. Results revealed authentic leadership to be the only significant predictor, *B* = 2.22, *SE* = .34, *t* = 6.47, *p* < .001, supporting the predicted effect of authentic leadership on employee team identification in multidisciplinary professional teams.

Study 1 provides support for the first stage of our theoretical model that authentic leadership has a positive causal effect on employee team identification in multidisciplinary professional teams. In the next study we continued to test the second stage of the model: the causal effect of team identification on OCB and the moderating role of professional identification in multidisciplinary professional teams.

### Results of Study 2

#### Manipulation check.

On a 7-point Likert scale (1 = *strongly disagree*, 7 = *strongly agree*), the participants reported the extent to which they identified with the team that they have recalled. The participants in the high team identification condition reported higher team identification (*M* = 5.78, *SD* = 1.20) compared to those in the low team identification condition (*M* = 3.21, *SD* = 1.67), *t*(149) = 10.85, *p* < .001.

#### Measurement model.

We employed AMOS [107] to assess whether the self-reported measures in the current study captured distinctive constructs. The results supported the validity of our measures based on CFI values close to one [108] as well as RMSEA and SRMR values of.08 or less [109]. Our full measurement model, including OCB, professional identification, professional differentiation, and professional salience produced χ^2^ = 375.56 (df = 183), p < .00 with indices that indicate acceptable fit (CFI = .93; SRMR = .06; RMSEA = .08). We compared this to two alternative models, each of which demonstrated comparatively poor fit. We investigated a model in which combined professional identification and professional differentiation which generated χ^2^ = 726.81 (df = 186), p < .00 suggestive of relatively poor fit (CFI = .79; SRMR = .12; RMSEA = .14). We then investigated a model in which professional identification and professional salience were combined which generated χ^2^ = 659.15 (df = 186), p < .00 suggestive of relatively poor fit (CFI = .82; SRMR = .13; RMSEA = .13). We also assessed a single factor model, which generated χ^2^ = 1475.59 (df = 189), p < .00) also suggestive of relatively poor fit (CFI = .51; SRMR = .19; RMSEA = .21). These results support the discriminant validity of our measures.

Descriptive statistics and correlations are presented in Table 2.

**Table 2:** Descriptive statistics and correlations among key variables in Study 2.

Variable	*M*	*SD*	2	3	4	5	6	7
1. Team identification	.50	.50	−.01	.25^**^	−.03	.04	.05	−.06
2. Professional identification	5.68	1.13		.35^**^	.20^*^	.23^**^	.10	.16^*^
3. OCB	5.10	1.41			.21^**^	.21^**^	.11	.24^**^
4. Professional differentiation	4.87	1.07				.45^**^	.16^*^	.05
5. Professional salience	5.14	1.34					.19^*^	.03
6. Age	37.25	9.89						−.06
7. Gender	.42	.51						

Note: ^*^ p < .05, ^**^ p < .01, ^***^ p < .001; team identification (0 = low, 1 = high); gender (0 = male, 1 = female).

#### OCB.

**Fig 2:**
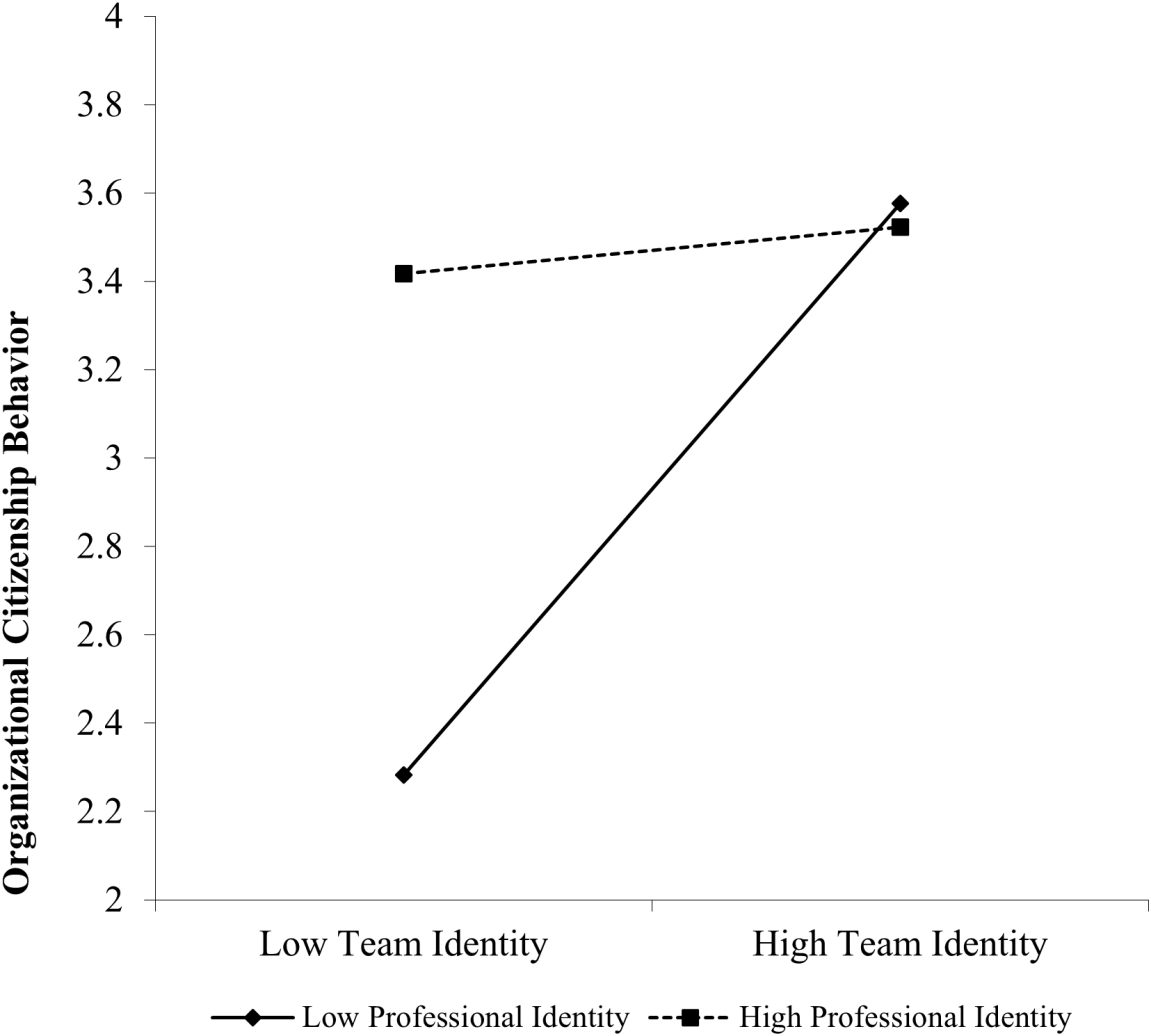
Professional identification moderates the effect of team identification on OCB in Study 2. The results of Study 2 provide support for the second causal link in our theoretical model, showing that team identification increases OCB and this causal effect is stronger when professional identification is low. Now that we have established the causal chain in the model, we aim to comprehensively test the whole model in a multi-source field survey in Study 3.

We used PROCESS (Model 1; [110]; all bootstrap analyses across all the studies used 5,000 resampling iterations) to test whether professional identification moderates the effect of team identification on OCB. Team identification and professional identification were centred. Participant age, gender, professional differentiation, and professional salience were standardized and included in the analysis as covariates. Results revealed a significant interaction between team identification and professional identification when predicting OCB, *B* = −.52, *SE* = .18, *t* = −2.94, *p* = .004. Specifically, when professional identification is low, team identification increases OCB (*B* = 1.19, *SE* = .25, *t* = 4.73, *p* < .001). In contrast, when professional identification is high, team identification does not influence OCB (*B* = .05, *SE* = .30, *t* = .17, *p* = .87. This pa*t*tern is illustrated in Fig 2.

### Results of Study 3

#### Measurement model.

The discriminant validity of our constructs was assessed using AMOS [107] to conduct a confirmatory factor analysis (CFA) with all latent variables measured in the members questionnaire. To assess measurement model fit we evaluated our results against the CFI value criteria of close to one [108] and RMSEA values of.08 or less [109]. This analysis generated χ^2^ = 608.57 (df = 293), p = .00 with fit indices that suggest acceptable fit to the data (CFI = .82; RMSEA = .05). Though the CFI result was lower than expected, it was above.80 and the more stringent RMSEA results supported model fit. We compared this against a three-factor model in which we combined authentic leadership and team identity. This analysis generated χ^2^ = 695.87 (df = 296), p = .00 with fit indices that suggest comparatively poor fit to the data (CFI = .78; RMSEA = .06; SRMR = .11). We compared these models against a single factor model, which generated χ^2^ = 1218.25 (df = 299), p = .00. The fit indices of this single factor model suggest poor fit to the data (CFI = .49; RMSEA = .09). These data provide support for the discriminant validity of our construct measures.

Descriptive statistics and correlations are presented in Table 3.

**Table 3:** Descriptive statistics and correlations among key variables in Study 3.

		*M*	*SD*	1	2	3	4	5
1	Professional Diversity	.60	.26					
2	Authentic Leadership	4.82	.63	.12				
3	Team Identification	5.09	.66	.16	.72^***^			
4	Professional Identification	5.40	.65	.25	.63^***^	.63^***^		
5	OCB	5.31	1.42	.16	.18	.11	.15	

Note: ^*^ p < .05, ^**^ p < .01, ^***^ p < .001.

Subsequent to assessing that regression assumptions were met and VIF figures were below 2.0 [111], we investigated our hypotheses using hierarchical regression analyses in PROCESS SPSS [110]. We retained team-level aggregation and utilized PROCESS, rather than multilevel modelling, as our ICC(2) results were similar to previous research in interprofessional healthcare teams [112], our ICC(1) results were acceptable, and our conceptualizations and operationalizations were at team level. We note that low ICC(2) figures suggest that our results may reflect conservative estimates of effects [98]. Table 4 reports the regression results relating to our hypotheses.

**Table 4:** Regression results for testing H1 to H4 in Study 3.

	H1: Team Identification	H2: Team OCB	H3: Team OCB	H4: Team OCB
Professional Diversity	.20 (.24)	.78 (.73)	.13 (.70)	1.14 (.70)
Authentic Leadership	.73 (.10)^**^			.16 (.42)
Team Identification (TI)		.19 (.29)	.07 (.34)	−.007 (.40)
Professional Identification (PI)			−.18 (.36)	−.22 (.38)
Interaction Variables				
TI X PI			−1.22 (.36) ^**^	−1.19 (.37)^**^
ΔR^2^				.002
R^2^	.52^***^	.03	.20^*^	.21^*^

Note: ^*^ p < .05, ^**^ p < .01, ^***^ p < .001. Tabled values are unstandardized parameter estimates (standard errors).

H1. Results support a positive relationship between authentic leadership and team identity, *B* = .73, *SE* = .10, *t* = 7.63, *p* < .001, *CI*_95%_ = [.54,.93].

H2. No support is found for a relationship between team identity and OCB, *B* = .19, *SE* = .29, *t* = .66, *p* = .51, *CI*_95%_ = [−.39,.76]).

**Fig 3:**
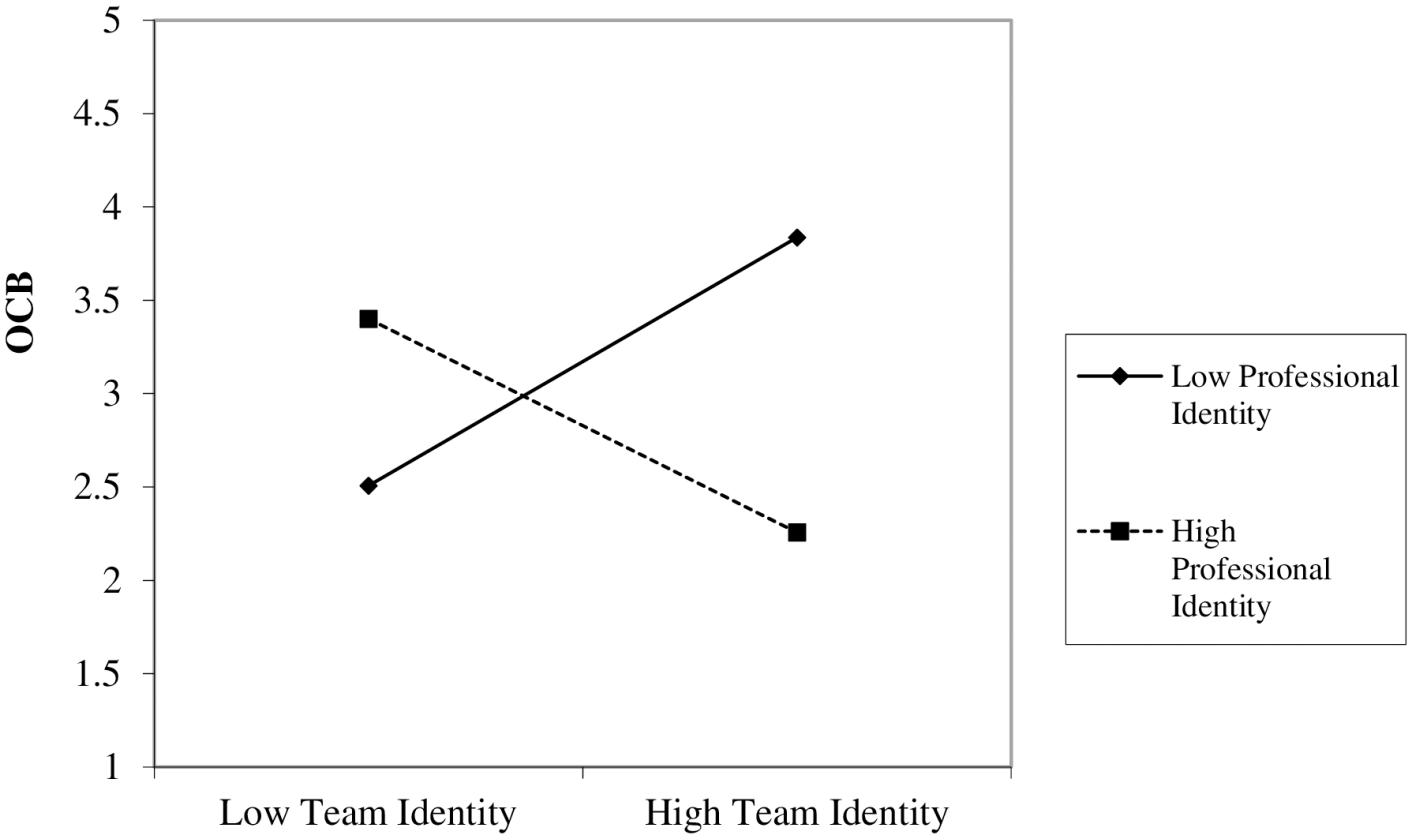
Professional identification moderates the effect of team identification on OCB in Study 3.

H3*:* We used Model 1 in PROCESS SPSS [110] with both predictors mean-centered [113] to test whether professional identity moderates the relationship between team identity and OCB. Results revealed a significant interaction, *B* = −1.22, *SE* = .36, *t* = −3.38, *p* = .001, *CI*_95%_ = [−1.94, −.50]). S*p*ecifically, results of a Johnson-Neyman significance region analysis showed that the effect of team identification on OCB was significantly positive when professional identification was below 4.71 (17% of our sample) and significantly negative when professional identification was above 6.04 (15% of our sample). The interaction pattern is depicted in Fig 3.

H4. We used Model 14 in PROCESS SPSS to investigate our fourth hypothesis (all predictors mean-centered). The results of testing the whole moderated mediation model showed that, when predicting OCB simultaneously, the interaction term between team identification and professional identification was the only significant predictor, *B* = −1.19, SE = .37, *t* = −3.19, *p* = .002, *CI*_95%_ = [−1.93, −.44], while all other predictors were not significant, *p*s > .11. Specifically, results of a Johnson-Neyman significance region analysis showed that the indirect effect of authentic leadership on OCB via team identification was significantly positive when professional identity was below 4.42 (12% of our sample) and significantly negative when professional identity was above 6.18 (10% of our sample), supporting a moderated mediation model, *I*_moderated mediation_ = −.87, *SE* = .32, *CI*_95%_ = [−1.55, −.25].

## General discussion

Three empirical studies converge to provide strong supporting evidence for our theoretically-driven moderated mediation model. Using a causal chain design, Studies 1 and 2 first established, in multidisciplinary professional teams, the positive causal effect of authentic leadership on team identification as well as the causal effect of team identification on OCB moderated by professional identification (stronger effect at lower levels of professional identification). Using a multi-source design in the field, Study 3 further provided comprehensive supporting evidence for the whole theoretical model. Together our theoretical model and the supporting findings offer important contributions to the leadership and citizenship literatures with subsequent significant managerial implications.

### Theoretical implications

We drew largely on social identity theory to highlight the capacity of authentic leaders to engender team identity in work teams. The results of both Study 1 and Study 3 support our contention that authentic leaders can foster the development of employee team identification. This finding should be interpreted in the context of past research that demonstrates the tensions that frequently arise in multidisciplinary professional teams such as multidisciplinary professional healthcare teams [114–117]. The potential for authentic leaders to engender team identification in such contexts is especially noteworthy.

Our studies move toward addressing the challenges faced by authentic leaders striving to build citizenship. An important contribution of our research stems from the finding that professional identification can help distinguish between the divergent effects of team identification and, through this, authentic leadership. Our results suggest that the citizenship benefits of team identification occur only when teams are characterized by weak professional identification, which lessens the potential tension between professional and team priorities. This finding provides a new perspective for us to interpret and understand the ambiguous results related to authentic leadership effects found in past reviews [20]; increasing team identification, authentic leaders’ capacity to increase citizenship behaviors is contingent on employees’ professional identification.

A range of perspectives have been harnessed to explain the ambiguous effects of authentic leadership on citizenship, however little progress has been made in identifying factors capable of explaining and predicting when such leadership is beneficial or not. Our findings suggest that professional identification is capable of such differentiation, which is particularly significant given the priority afforded professional identification in healthcare [118,119] and thus for HR practitioners, especially in health services organizations. While past research has identified factors that negatively impact citizenship behavior, these tend to be dysfunctional organizational attributes such as poor leadership or dysfunctional coworker dynamics (e.g., [120,121]). Our findings suggest that factors that are traditionally considered to be positive (e.g., employee professional identification) might also attenuate or even reverse the positive effect of authentic leadership on OCB.

Our studies also contribute to the social identity approach by broadening our understanding of its influence on citizenship in teams. We argue that team identification does not universally enhance such extra-role behaviors, and may also paradoxically decrease citizenship (e.g., in healthcare contexts in Study 3), suggesting that the impact of team identification on citizenship behavior in teams is neither simple nor straightforward. While strong team identification may increase the likelihood that members will engage in citizenship behavior, we found that this effect is less likely to occur among employees who also identify highly with their professions. This indicates that the potentially destructive effects of perceived team dominance over professional concerns may undermine the benefits of strong team identification [78]. Support for our argument that competition between two types of identity may evoke negative behaviors represents an important extension to OCB theorizing. Especially in healthcare contexts, reflected in our field study, our findings suggest that increasing team identification may potentially even decrease citizenship behaviors in teams where members identify strongly with their respective professions. This, in turn, has significant implications for organizational leaders and literature.

### Implications for practice

In addition to our theoretical contributions, our findings have substantial practical implications. Though citizenship is often neglected in studies of healthcare teams [3], it remains of vital importance consequent to its patient, clinician and organizational benefits [122–124]. The potential for authentic leaders to enhance both team identification and citizenship thus has significant implications for healthcare managers and also reinforces the importance of authenticity for practicing HR professionals.

Although some employees may not require such support, many potential leaders may gain from interventions that provide the opportunity to develop their authentic leadership capacity. Following Nübold et al. [125], we suggest the value of mindfulness interventions which have been demonstrated to elevate authentic leadership potential. Equally important, the ability of organizations to develop authentic leaders relies on the avoidance of characteristics that can undermine such leadership potential including personal insecurity [126]. Organizational development programs that aim to heighten self-awareness through mindfulness and strategies that limit the emergence of toxic cultures dominated by those with insecure attachment styles [125–127] are likely to support the emergence of authentic leadership as are coaching interventions directed to build such leadership [14].

Our findings also indicate that the capacity of authentic leaders to enhance OCB is likely to depend upon their capacity to engender team identification while also limiting professional identification. Although the normative consensus in the field is to engender team identification in order to achieve positive team outcomes [128], our findings suggest that we should also take into consideration employees’ professional identification and address the concerns team members might have regarding the potential conflict between the different values, priorities and practices that the team and the profession might have. If HR interventions designed to support team identification fail to address this main concern from team members who identify strongly with their professions, they might have no or even a dysfunctional effect on extra-role behavior.

This is particularly relevant given consistent findings that strong professional identification characterizes professional bureaucracies, and is particularly influential in healthcare [129]. This insight suggests caution by HR practitioners and researchers alike, and attention to precisely under what circumstances well intended initiatives designed to invoke team identification are actually effective in increasing employee citizenship behaviors. Further, our findings suggest merit in considering a more complex approach in which both team and profession are recognized as equally significant, and interactive, social categories.

This has several implications for HR management, particularly in professional bureaucracies in which professional identity may be relatively well established. Authentic leaders are advised to build team identification by, for example, drawing team members attention to the value of their collective and diverse contributions [22,130]. Pairing such strategies with efforts to minimize the impact of professional identification, such as through strengthening superordinate identification [56,77], are also recommended by our findings. In alignment with the mutual intergroup differentiation model, attending to both team and profession as salient social identities is likely to provide authentic leaders with greater opportunity to enhance citizenship [131]. That said, such initiatives should be approach cautiously given the risks associated with perceived threat to professional identity [77,78,119]. In this context, we note that our analysis suggests that our model applies only to those teams with substantially high or low professional identification. It follows that between one-fifth and one-quarter of teams are likely to be influenced by the relationships that we model, which, we argue, represents a significant proportion of teams. However, leaders should be cognizant of this pattern when deciding which initiatives are likely to be most useful.

## Limitations and conclusion

A number of limitations should be acknowledged in considering our findings. First, while Study 2’s results suggest that increasing team identification when teams are characterized by high professional identification is unlikely to influence OCB, Study 3 showed that strengthening team identification in the same context may indeed decrease OCB. We posit that the differences in our field and experimental findings may be due to the critical role of profession in healthcare [112,119,132], and acknowledge that the healthcare context may represent an important boundary condition for our model [133]. Previous research suggests that profession is prioritized in healthcare above most other social categories and that high professional identification may motivate efforts to protect professional autonomy, status and distinctiveness [119,134]. Our field results suggest that this effect may diminish the impact of team identification on OCB as members focus on advancing their own profession’s priorities. However, we also posit the centrality of profession may be likely in other professional bureaucracies, suggesting the generalizability of our model well beyond healthcare environments. Future research in professional industries beyond healthcare can test this possibility. We also note the US national context of our research as a further boundary condition and acknowledge that, while our results may be applicable to similar health systems, future research in non-OECD countries and emerging economies would substantially strengthen generalizability.

Second, notwithstanding the strengths of the recall method in capturing participants’ authentic workplace experiences, we acknowledge its inherent limitations. Because recall designs rely on participants’ retrospective accounts, they may be subject to memory bias or post-hoc rationalization. To mitigate these risks, we provided clear instructions and used validated scales to guide participants’ reflections. Nonetheless, future research could extend this work by incorporating complementary approaches, such as vignette-based or longitudinal designs, to further enhance methodological rigor and strengthen the robustness of causal inferences. Furthermore, the sample size and low ICC(2) results in Study 3 potentially limit our capacity to identify significant relationships [135], and our results may reflect a conservative estimate of true team-level relationships. Our hypotheses were largely supported and confirmed in experimental studies, however we acknowledge the value of replication using larger field samples. Such future research may also endeavor to address issues of endogeneity that may have been present in study 3, though our experimental results provide confidence in the robustness of our findings.

We also note that field data for our predictor, mediator and moderator variables were collected from the same survey, which may lead to common method bias. While these methods are less likely to bias the complex moderated mediation relationships in our model [136], we suggest that future field research could address this limitation through the use of, for example, more objective observational measures of leadership. The use of such measures would also address our reliance on observational OCB measures and self/leader ratings.

Despite these issues, our research provides a signification contribution to our understanding of authentic leadership and the capacity of such leaders to enhance engender OCB. In particular, the capacity of a single moderator, professional identification, to account for inconsistency in the effects of authentic leadership, and team identification, provides insight into past ambiguous results, a valuable direction for future studies and useful insights for HR managers and organizational leaders interested in team-based work design.
